# TREM2 Is a Prognostic Biomarker and Correlated with an Immunosuppressive Microenvironment in Thyroid Cancer

**DOI:** 10.1155/2022/1807386

**Published:** 2022-11-16

**Authors:** Jing Wang, Zhendong Li

**Affiliations:** Department of Head and Neck Surgery, Cancer Hospital of China Medical University, NO.44 Xiaoheyan Road, Dadong District, Shenyang 110042, Liaoning Province, China

## Abstract

**Purposes:**

To identify the differentially expressed genes (DEGs) related to the immune microenvironment and elucidate the biological functions of key genes in papillary thyroid cancer (PTC) by analyzing the immune microenvironment.

**Methods:**

The relative quantities of immune and matrix components in 507 patients with PTC were calculated from the TCGA database. Analysis of differentially expressed genes in tumor samples throughout the genome, intersection of DEGs obtained from PTC patients, and genome-wide tumor samples and survival analysis were performed. Survival analysis was used for identification of prognostic factor. Immunohistochemical analysis of the TREM2 expression in PTC tissues, flow cytometry, and transwell assays were used to detect the effect of TREM2 on PTC cell proliferation, migration, and invasion.

**Results:**

There were a total of 1242 upregulated genes with high intersection in the immune score and 124 downregulated genes with low intersection in the stromal score. A total of 1,366 genes in these DEGs may be determinants in the immune microenvironment. GO enrichment and KEGG enrichment analysis revealed that the overall function of DEGs appeared to map onto immune-related activities. Gene intersection and survival analysis showed that there were 435 DEG crosses in PTC patients and genome-wide tumor samples, only *CXCL10*, *CD40LG*, *KRT14*, *TRAT1*, and *TREM2* were associated with patient prognosis, and TCGA showed that only the *TREM2* expression was upregulated in PTC. *TREM2* knockdown inhibited the cell cycle and cell proliferation, migration, and invasion by PTC cells. TREM2 was associated with the immunosuppressive microenvironment by via NF-*κ*B pathway in PTC.

**Conclusion:**

TREM2 possibly was a potential indicator of altered TME status in PTC, and that TREM2 promoted PTC cell proliferation and cell cycle, migration, and invasion by NF-*κ*B pathway.

## 1. Introduction

Thyroid cancer is the most common endocrine malignancy globally. According to the Global Cancer Registry study, the incidence of thyroid cancer in many countries and regions of the world in recent years has been increasing annually [1]. A study showed that thyroid cancer is the fourth most common tumor in women in Eastern China [2]. Thyroid cancer is divided into several subtypes, including papillary thyroid carcinoma (PTC), follicular thyroid carcinoma, medullary thyroid carcinoma, and undifferentiated thyroid carcinoma [3–5]. The traditional treatment for PTC is surgery, and while the prognosis of patients with early-stage PTC is good, the 5-year survival rate for patients with advanced PTC is only about 59%. Tumor immunotherapy is a new treatment modality in PTC [6, 7].

Tumor immunity has been a hot topic of research in recent years [8]. Tumor cells can evade immune surveillance, immune recognition, and immune attack by the immune system through certain mechanisms, thereby preventing the body from producing an effective antitumor immune response [9, 10]. The tumor microenvironment (TME) is the environment in which tumor cells grow and develop and is composed of structural components, mainly stromal cells and immune cells [11, 12]. TME plays an important role in the development of tumors and is involved in malignant transformation, tumor growth, metastasis, and other processes [13, 14]. Tumor immunity focuses on the interaction of tumor cells with various immune cells, including T cells, dendritic cells (DCs), and macrophages in the TME. In recent years, immunotherapy has become the most promising treatment for tumors after surgery, radiotherapy, chemotherapy, and molecularly targeted therapy. Tumor immunotherapy controls and kills tumor cells by stimulating and enhancing the body's immune function [15]. Devising newer and more effective treatments for PTC has great relevance for basic and clinical translational research; thus, seeking novel anti-tumor immune molecules that enhance the body's anti-tumor immune response by altering the TME is an urgent unmet need.

Trigger receptor 2 (TREM2) is a recently identified immunoglobulin superfamily receptor that is selectively expressed in myeloid cells and undergoes intracellular signaling through DNAX-activated protein 12 and phosphorylated tyrosine kinase [16]. TREM2 binding to its ligand promotes high expression of CD163 in tumor-associated macrophages, amplifying the inflammatory response in the local TME and promoting tumorigenesis and tumor progression [17]. Recent studies have identified TREM2 as an emerging therapeutic target for cancer immunotherapy. For example, TREM2 deficiency and anti-TREM2 monotherapy caused significant changes to the macrophage population infiltrating the tumor, with reduced infiltration of MRC1 and CX3CR1-labeled immunosuppressive macrophages and an expansion of a new subpopulation expressing immunostimulatory molecules, with greater numbers of PD-1-expressing CD8+ T cells and CD4+ T cells in the tre2-/- tumor-infiltrated group compared to the wild type [18]. In addition, it was suggested that genetic excision of TREM2 significantly inhibited the accumulation of myeloid cells within the tumor, leading to immune reactivation [19]. These results imply that TREM2 may play an important role in tumor immunity. However, the role of TREM2 in PTC is as yet unclear. In view of the above research lacuna, in the current study, the differentially expressed genes (DEGs) generated from the comparison of the immune and stromal components of PTC were used as a starting point to elucidate through a series of analyses that possibly TREM2 is a potential indicator of altered TME status in PTC; we also sought to unravel the biological role of TREM2 in PTC, providing new perspectives and directions for the diagnosis and treatment of PTC.

## 2. Materials and Methods

### 2.1. Data Preparation and Scoring

Transcriptomic RNA-seq data of 507 PTC cases and corresponding clinical data (age, gender, tumor grade, pathological stage, and survival outcome) were retrieved from the TCGA database and normalized using limma software package. Normal or duplicate samples were removed. The proportion of the immune and stromal constituents of the TME was then estimated for each sample using the *R* language 4.0.2, set with the ESTEST software package, and expressed as two scores, the immunity score and the stroma score, respectively, which were closely connected with the ratio of immune cells, stromal cells, and their total value; meaning, thereby that the higher the score, the greater the ratio of the relevant constituent within the TME.

### 2.2. Survival Analysis and Extraction of DEGs

With the help of the software package survminer, survival analysis was conducted. Of 507 tumor specimens, 421 had detailed survival times recorded for survival analysis. Immediately afterwards, based on comparison with the median immune score and stromal score, the 507 tumor samples were labeled as high or low subgroups, respectively. Differential analysis of the gene expression was conducted by utilizing the limma software package by comparing DEGs obtained from high and low scoring samples. DEGs with an absolute value of log_2_ (fold change) > 1.0 (high subgroup/low subgroup) and FDR < 0.05 were recognized as statistically important.

### 2.3. Gene Ontology (GO) and Kyoto Encyclopedia of Genes and Genomes (KEGG) Analyses

The obtained DEGs were subjected to GO and KEGG analyses through the *R* packages clusterProfiler, enrichplot, and ggplot2, and pathways with *P* values and *q* values <0.05 were recognized to be obviously rich. Heat maps of DEGs were generated using the *R* language in combination with the pheatmap package, after which data related to clinicopathological features corresponding to PTC specimens were obtained from TCGA. Statistical analysis was conducted using the *R* language; the Wilcoxon rank sum test or Kruskal-Wallis rank sum method was used for comparison between different clinical stages.

### 2.4. Clinical Samples

In this study, tissues from 20 patients with thyroid cancer and 17 patients with benign thyroid tumors admitted to our hospital between January 2018 and February 2019 were collected. Inclusion criteria were as follows: (i) diagnosis of thyroid cancer was made on the basis of the diagnostic criteria in the 2015 NCCN guidelines, (ii) age ≤ 79 years, (iii) diagnosis was confirmed by histopathological examination;, and (iv) no history of radiotherapy or endocrine treatment in patients. Exclusion criteria were as follows: (i) history of other thyroid surgery, (ii) coexistence of other malignant tumors, (iii) patients with immune function disorders, and (iv) missing pathological data.

### 2.5. Immunohistochemistry

Thyroid cancer paraffin tissue microarrays were dewaxed and hydrated and then remove endogenous peroxidase, and diaminobenzidine (DAB) was used for color development; nuclei were stained with hematoxylin staining solution; sections were dehydrated till transparent, sealed, and observed under the microscope. The mean numbers of positive and strongly positive cells in each group were calculated and compared.

### 2.6. The qRT-PCR

Total RNA was extracted from PTC cells using the TRIzol method. *TREM2* was reverse-transcribed using the stem-loop method and the common reverse transcription method to obtain first-strand cDNA. The qRT-PCR was performed using the miR-XTM miRNA qRT-PCR SYBR Kit and SYBR Green dye. TREM2 was treated with GAPDH as an internal reference. We referred to the literature for reaction conditions and primer sequences. TREM2 F: 5′-GGAGCACAGCCATCACAGAC-3′, R: 5′-CACATGGGCATCCTCGAAGC-3′. GAPDH: F: 5′-GTCTCCTCTGACTTCAACAGCG-3′, R: 5′-ACCACCCTGTTGCTGTAGCCAA-3′.

### 2.7. Western Blot

Because the cells had been processed, the medium was discarded; protein lysate (Roche) was added, and total protein was separated. An aliquot of 50 g of total protein was spiked on a 12% polyacrylamide gel and subjected to electrophoresis under 100 V for 120 min. The membrane was then transformed to a PVDF film (Millipore, USA). Then, 5% powdered skim milk was closed for 1 h at room temperature, membranes were washed thrice with TBST for 10 min each time, and the closed films were incubated with primary antibody (concentration 1 : 1,000) at 4°C overnight. The membranes were then incubated with HRP-labeled anti-rabbit secondary antibody (1 : 3,000) under ambient temperature for 60 min. The membranes were washed thrice with TBST for 600 s each time. Finally, the films were imaged with western blot special reagents (Invitrogen, Carlsbad, CA, USA) for color development, and the grey values of each protein were analyzed.

### 2.8. Cell Culture and Transfection

Human thyroid cancer SW579 and KTC-1 cells and human normal thyroid epithelial cells Nthy-ori 3-1 were bought from Cell Bank of Chinese Academy of Sciences (Shanghai). Cells were cultivated in a sterile incubating device under 37°C, 5% CO_2_, and 5% v/v. TREM2 siRNA and its negative control (NC) were constructed by GenePharma (Shanghai, PRC). Blank plasmids were used as controls. SW579 cells at logarithm growth phase were inoculated in 6-well dishes and transfected with control, NC, and TREM2 siRNAs at 70%–80% cell fusion after following the instruction manual of Lipofectamine 2000 (Invitrogen; Thermo Fisher Scientific, Inc.). After 48 h of transfection, total RNA was obtained, and RT-PCR was conducted to demonstrate the efficiency of transfection.

### 2.9. Flow Cytometry

The cells were inoculated in 96-well plates at 1 × 10^4^ cells/well, incubated for 24 h, washed twice in PBS, subjected to fixation in 70% ethyl alcohol, and incubated overnight at 4°C. Cells were washed once in PBS, and the cellular density was changed to 1 × 10^6^ cells/mL. Propidium iodide dyeing liquor was added to an eventual amount of 0.05 mg/mL, and cells were stained for 30 min at 4°C. The cell cycle in all groups was analyzed by flow cell technique. Three replicate wells were set up for all groups, similar tests were repeated three times, and the mean result was taken.

### 2.10. Transwell Assay

Cells were inoculated in 24-well plates equipped with small chambers, with two replicate wells set up for each group. Cells were stopped in serum-free intermediary in the upper chamber (approximately 10^5^ cells/well) (8 *μ*m pore size; BD Biosciences), and 500 *μ*L of DMEM intermediary involving 10% FBS was added to the lower chamber. After 24 h, cells on the upper chamber surface (i.e., cells that had not entered into the lower chamber) were lightly swabbed with a swab, and cells invading the lower chamber were stabilized in methyl alcohol and then dyed in crystal violet. Five randomly selected areas of view (including the center and periphery of the membrane) were visualized under an inverted microscope, and the number of cells invading the lower chamber was calculated. The bottom of the transwell chamber was covered with Matrigel for the invasion assay, and the other procedures were similar to the migration assay.

### 2.11. Statistical Analysis

Statistical analysis was done using SPSS 22.0 software package (USA). Measures are shown as the average ± standard deviation ($\bar{x}\pm s$); the *t*-test was used to compare sample means between groups; one-way ANOVA was used for comparing several sample means, and SNK-q test was used for two-way comparison between multiple sample means. Statistical significance was set at *P* < 0.05.

## 3. Results

### 3.1. DEGs Correlated with the Immunosuppressive Microenvironment in Thyroid Cancer

**Figure 1:**
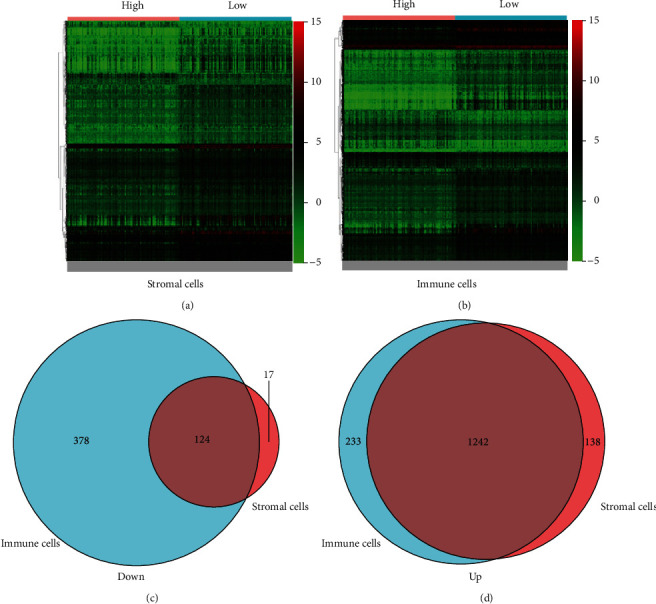
DEGs correlated with immunosuppressive microenvironment in thyroid cancer. (a) Analysis of differential genes and segregation into two groups (high and low groups) based on stromal cell scoring. (b) Analysis of differential genes and segregation into two groups (high and low groups) based on immune cell scoring. (c) Intersection of upregulated genes. (d) Intersection of downregulated genes.

**Figure 2:**
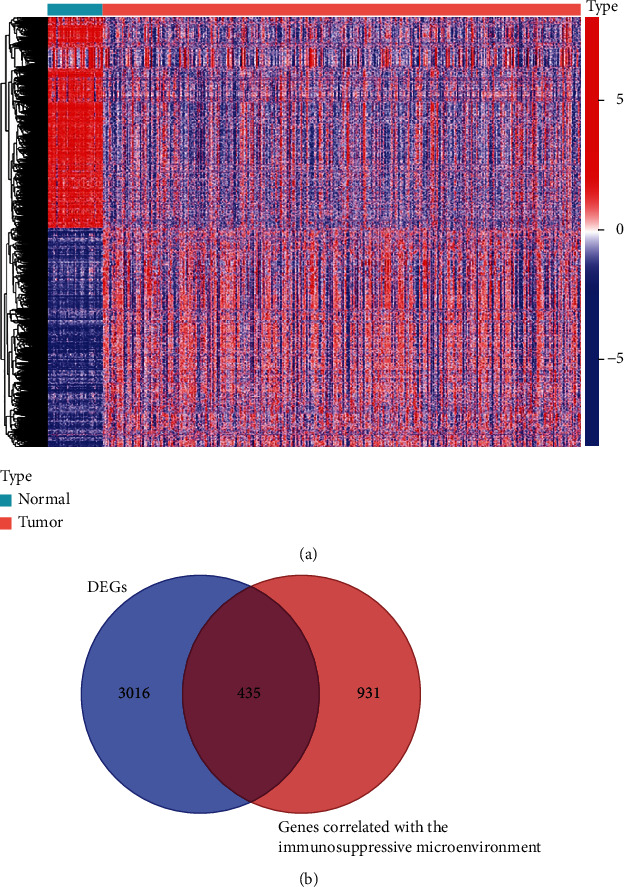
Intersection of DEGs obtained from PTC patients and genome-wide tumor samples. (a) Genes differentially expressed in tumor samples throughout the genome. (b) In the whole genome, 3451 genes were differentially expressed in tumor samples, which intersected with 1366 genes in Figure 1, and 435 genes were obtained.

A comparative analysis of high and low scoring samples was performed in order to determine the exact variation in gene profiles in the immune microenvironment with respect to immune and stromal components. Compared to the median, 1,521 DEGs were obtained from the stromal score (1,380 upregulated genes and 141 downregulated genes (Figure 1(a)). Similarly, 611 DEGs were obtained from the immune score (high versus low scoring samples), with 233 genes upregulated and 378 genes downregulated (Figure 1(b)). Intersection analysis revealed a total of 1,242 upregulated genes from the immune score and 124 downregulated genes from the stromal score with high intersection and 124 genes with low intersection (Figures 1(c) and 1(d)). A total of 1,366 genes in these DEGs could be determinants of the immune microenvironment. Besides, the transcriptome data of thyroid cancer were downloaded from The Cancer Genome Atlas (TCGA) database. The results showed that 3451 genes were differentially expressed in tumor samples in the whole genome, and 435 genes were obtained by intersection with 1366 genes in Figures 2(a) and 2(b).

### 3.2. Gene Ontology (GO) and Kyoto Encyclopedia of Genes and Genomes (KEGG) Analyses of DEGs Showed Correlation with the Immunosuppressive Microenvironment in Thyroid Cancer

**Figure 3:**
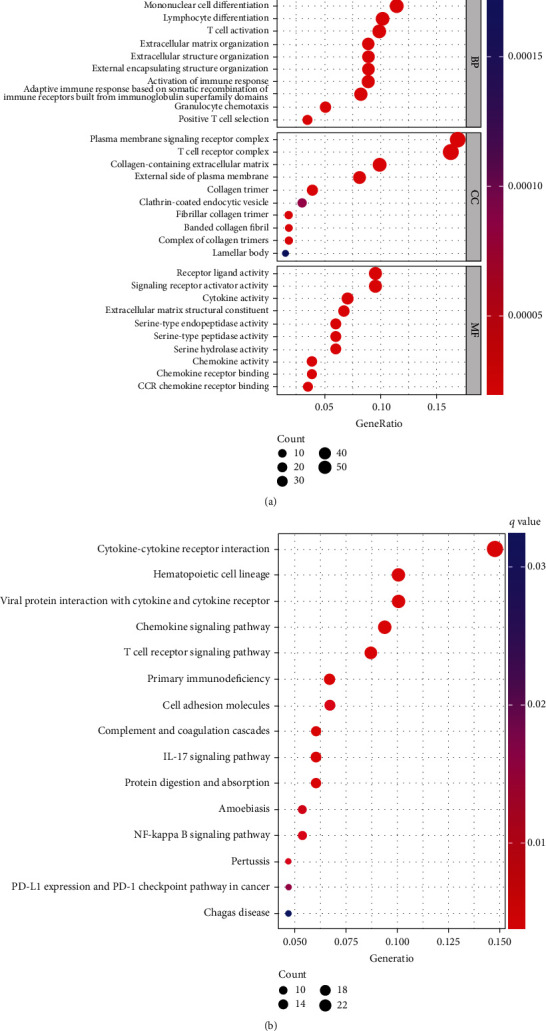
GO and KEGG analyses of DEGs showed correlation with immunosuppressive microenvironment in thyroid cancer. (a) GO analysis. (b) KEGG analysis.

**Figure 4:**
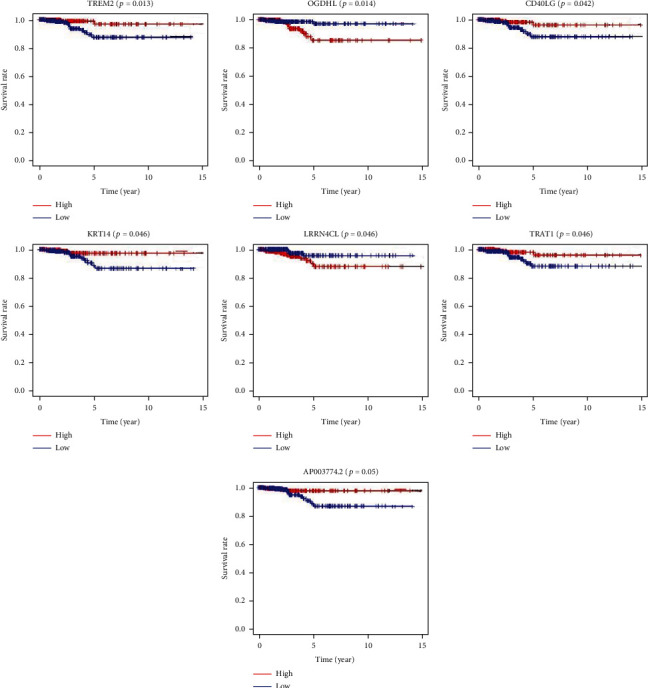
Survival analysis of PTC patients: DEGs correlated with the immunosuppressive microenvironment in thyroid cancer. Go analysis and KEGG analysis of 435 DEGs genes. Finally, only *CXCL10*, *CD40LG*, *KRT14*, *TRAT1*, and *TREM2* were associated with the survival rate in PTC patients.

Based on Go analysis and KEGG analysis of 435 genes, the results of the GO enrichment analysis showed that DEGs almost mapped to immune-related GO pathways, such as leukocyte proliferation and humoral immune responses (Figure 3(a)). KEGG enrichment analysis also revealed enrichment for primary immunodeficiency, cytokine-cytokine receptor interactions, and hematopoietic cell lineages (Figure 3(b)). Thus, the overall function of DEGs appeared to map onto immune-related activities, thereby suggesting that immune factors may be a potential feature of the immune microenvironment in thyroid cancer. Survival analysis of thyroid cancer patients was performed to identify significant factors affecting survival. Only *CXCL10*, *CD40LG*, *KRT14*, *TRAT1*, and *TREM2* were associated with patient prognosis (Figure 4).

### 3.3. TREM2 Was Upregulated in Thyroid Cancer

**Figure 5:**
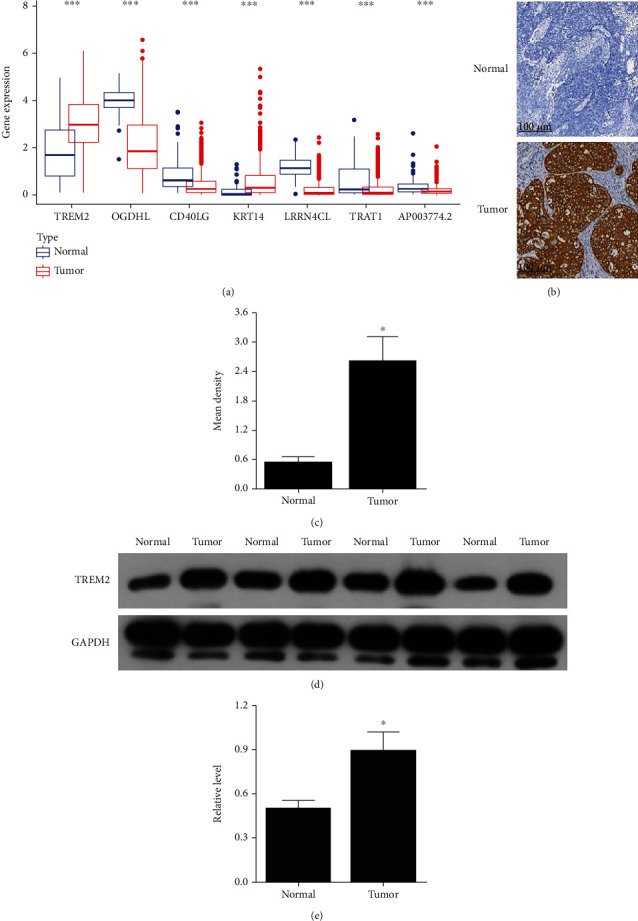
*TREM2* was upregulated in thyroid cancer. (a) Expression of *CXCL10*, *CD40LG*, *KRT14*, *TRAT1*, and *TREM2* in TCGA; we finally selected *TREM2* for subsequent validation. (b, c) Immunohistochemistry detection of TREM2 expression in thyroid cancer tissues. (d, e) Western blot detection of TREM2 expression in PTC tissues. ^∗^*P* < 0.05 vs. normal.

Subsequently, the TCGA database results showed the expression of *CXCL10*, *CD40LG*, *KRT14*, *TRAT1*, and *TREM2*, with only *TREM2* expression levels being upregulated in thyroid cancer (*P* < 0.05, Figure 5(a)). We finally selected TREM2 for subsequent validation. Similarly, the immunohistochemical results showed that the TREM2 expression was mainly localized to the cytoplasm and was expressed in both thyroid cancer tissues and controls, with a significantly higher expression in cancer tissues than in controls. The mean number of positive cells in the cancer tissues was significantly higher than that in the control group (*P* < 0.05, Figures 5(b) and 5(c)). Western blot results showed that the TREM2 expression was upregulated in thyroid cancer tissues (*P* < 0.05, Figures 5(d) and 5(e)).

### 3.4. TREM2 Knockdown Inhibited Cell Cycle and Cell Proliferation and Migration and Invasion by SW579 and KTC-1 Cells

**Figure 6:**
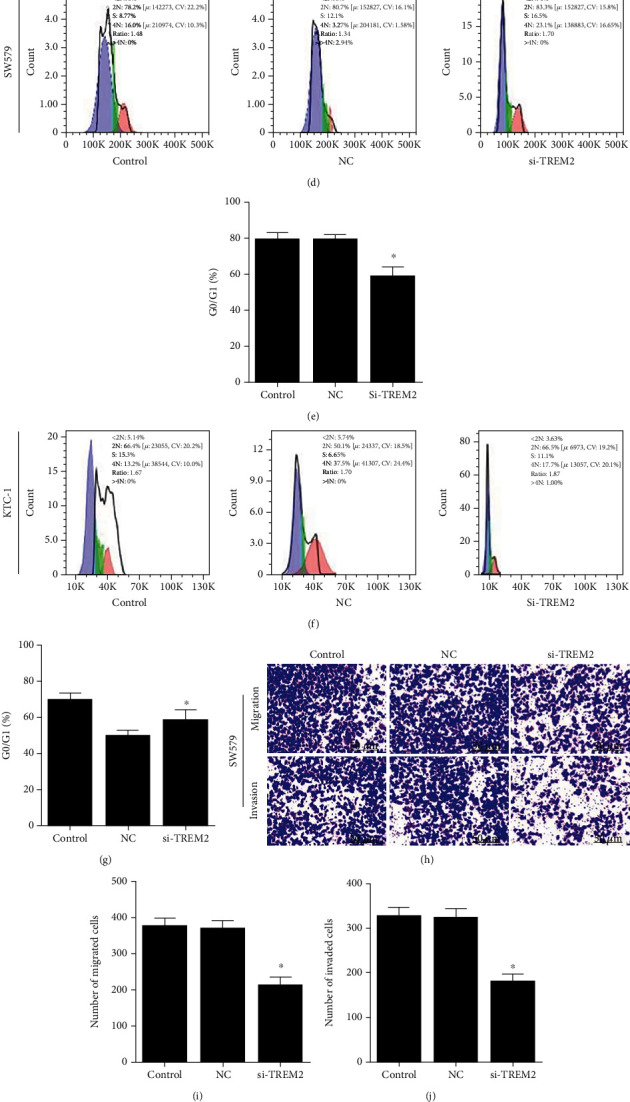
*TREM2* knockdown inhibited cell cycle and proliferation, migration, and invasion by SW579 and KTC-1 cells. (a) qRT-PCR detection of TREM2 expression in thyroid cancer cells. (b) Detection of knockdown efficiency of si-TREM2 in SW579 and KTC-1 cells. (c, d) Western blot detection of TREM2 expression. (d)–(g) Flow cytometry detection of cell proliferation. (h)–(m) Transwell assay of cell migration and invasion ability. ^∗^*P* < 0.05 vs. NC.

The qRT-PCR results indicated that TREM2 was significantly raised in thyroid cancer cells (*P* < 0.05, Figure 6(a)). Later on, the effects of si-TREM2 on PTC cell proliferation, cell cycle, cell migration, and invasion were assessed. qRT-PCR showed that TREM2 was significantly reduced by siRNA, and siRNA 2 obtained higher efficiency (*P* < 0.05, Figure 6(b)). siRNA 2 was selected for the experiment. Western blot result showed that si-TREM2 significantly decreased TREM2 in SW579 and KTC-1 cells (*P* < 0.05, Figure 6(c)). Flow cytometry results suggested that G0/G1 was downregulated in the si-TREM2 group compared to the control group (*P* < 0.05, Figures 6(d)–6(g)), thereby suggesting that si-TREM2 was able to inhibit cell activity and the cell cycle in SW579 and KTC-1 cells. In addition, the results of transwell assay showed that the extent of migration and invasion of SW579 and KTC-1 cells was inhibited in the si-TREM2 group (*P* < 0.05, Figures 6(h)–6(m)). These results suggested that *TREM2* knockdown inhibited the cell cycle and proliferation, migration, and invasion of SW579 and KTC-1 cells.

### 3.5. TREM2 Knockdown Involved in Immune Escape of Thyroid Cancer Cells via NF-*κ*B Pathway

**Figure 7:**
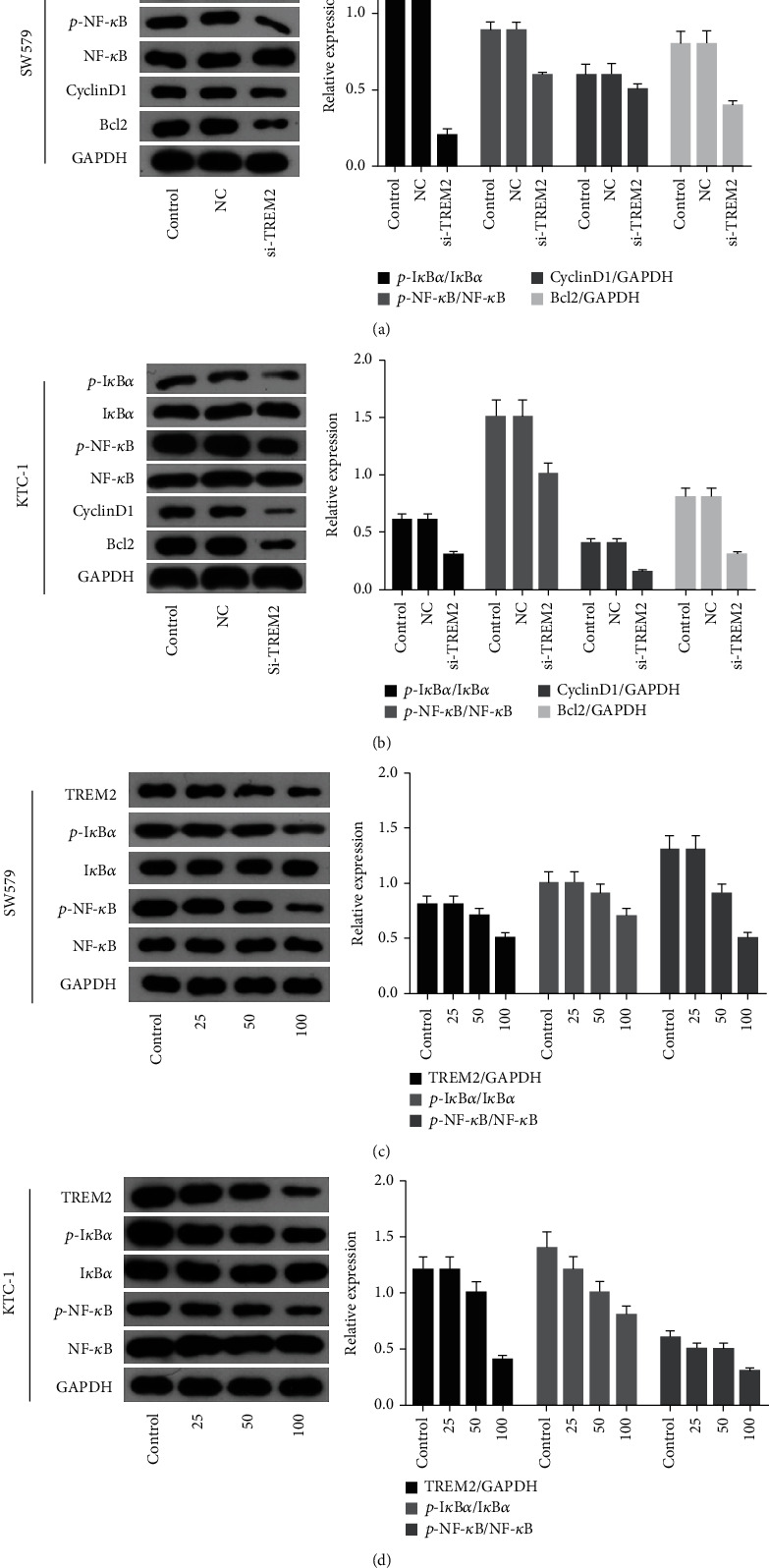
TREM2 knockdown involved in immune escape of thyroid cancer cells via NF-*κ*B pathway. (a, b) The expression of I*κ*B-*α*, p-I*κ*B-*α*, NF-*κ*Bp65, and p-NF-*κ*Bp65 in SW579 and KTC-1 cells was detected by western blot. (c, d) Detection of p-NF-*κ*Bp65, p-I*κ*B-*α*, and TREM2 levels after blocking the expression of NF-*κ*B protein using different concentrations of PDTC. ^∗^*P* < 0.05 vs. control.

It has been reported that nuclear transcription factor- (NF-) *κ*B consists of five subunits, including NF-*κ*B p65, and that NF-*κ*Bp65 is activated in response to pathogenic factors, while tumor tissue T cells are activated to secrete large amounts of interleukin (IL)-6, IL-10, and tumor necrosis factor (TNF)-*α*, all of which have immunosuppressive effects and are thus involved in the immune escape of malignant tumor cells [20]. To further investigate the mechanism of TREM2 promoting thyroid cancer cell activity, we used western blot to detect the expression of I*κ*B-*α*, p-I*κ*B-*α*, NF-*κ*Bp65, and p-NF-*κ*Bp65 genes in SW579 and KTC-1 cells. The results showed that si-TREM2 inhibited the phosphorylation of NF-*κ*Bp65 and I*κ*B-*α* proteins and significantly downregulated the expression of p-NF-*κ*Bp65 and p-I*κ*B-*α*. It also downregulated the expression of proliferation-related protein cyclin D1 and antiapoptosis-related protein Bcl2 in the downstream pathway (*P* < 0.05, Figures 7(a) and 7(b)). This suggests that TREM2 plays a pro-proliferative role in thyroid cancer cells by activating the NF-*κ*B signaling pathway and upregulating the expression of cyclin D1 and Bcl2; after blocking the expression of NF-*κ*B protein using different concentrations of the small molecule inhibitor pyrrolidine dithiocarbamate (PDTC) (25-100 *μ*M), the expression of p-NF-*κ*Bp65 and p-I*κ*B-*α* protein was significantly downregulated as the PDTC dose gradually increased. The expression of TREM2 protein was also decreased after blocking the NF-*κ*B pathway (*P* < 0.05, Figures 7(c) and 7(d)).

## 4. Discussion

PTC is the most common subtype of thyroid cancer but is difficult to cure [21] because of the lack of effective therapeutic targets and molecular agents. Over the past 12 years, medical treatment of PTC has transitioned from targeting of nonspecific immune pathways (the cytokine era) to targeted therapies against vascular endothelial growth factor (VEGF) and now to new immunotherapeutic agents [22, 23]. Therefore, better patient selection so as to optimize response to specific drugs remains a major challenge. In this study, a total of 1,366 intersecting DEGs were screened after a comparison of high and low immune and stromal scoring groups. GO analysis and KEGG pathway analysis showed significant aggregation of DEGs in cytokine-cytokine receptor interactions and primary immune defects, suggesting a close association with the immune microenvironment.

TREM2 is a signaling receptor present on the surface of cell membranes and plays a key role in pathological processes, such as immune response and inflammatory response. It has been found that the abnormal TREM2 expression was associated with the development and progression of neurological pathologies, such as Alzheimer's disease [24]. Tang et al. showed that TREM family members induced biological behaviors, such as proliferation, invasion, and migration of hepatocellular carcinoma cells [25]. Hamerman et al. found that TREM2 bound to the junction protein DAP12 and activated PI3K and inhibited TLR activation, which in turn inhibited tumor necrosis factor secretion and macrophage activity; inhibition of tumor necrosis factor secretion and macrophage activity was closely related to tumorigenesis and tumor progression [26]. Melchior et al. reported that TREM2 inhibited T cell proliferation and negatively regulated tumor immunity [27]. Binnewies et al. concluded that the high TREM2 expression amplified the inflammatory response in the TME and promoted tumor cell formation, and that the local inflammatory microenvironment also mediated epithelial-mesenchymal phenotype transformation and promoted tumor cell invasion and migration [28]. Therefore, TREM2 may be involved in tumorigenesis through mechanisms, such as induction of damage and regulation of tumor immunity. In the current study, there were a total of 1,242 upregulated genes with high intersection in the immune score and stromal score and 124 downregulated genes with low intersection. A total of 1,366 genes among these DEGs may be determinants of the immune microenvironment. In the whole genome, 3451 genes were differentially expressed in tumor samples, which intersected with 1366 genes in Figure 1, and 435 genes were obtained. The results of GO and KEGG analyses showed that the overall function of DEGs appeared to map onto immune-related activities, thereby suggesting that the involvement of immune factors may be a potential feature of the TME in thyroid cancer. In addition, survival analysis was used to identify significant factors affecting the survival of patients with thyroid cancer. 435 genes were analyzed for survival, and only CXCL10, CD40LG, KRT14, TRAT1, and TREM2 were associated with patient prognosis. Subsequently, TCGA database results showed the expression of CXCL10, CD40LG, KRT14, TRAT1, and TREM2, with only TREM2 expression levels being upregulated in thyroid cancer. We eventually selected TREM2 for subsequent validation.

Tumorigenesis is a continuous and complex process involving multiple factors, steps, and links. Epigenetic phenomena are one of the important factors causing tumor cell deterioration, including DNA methylation, ubiquitination, and histone modification [29, 30]. TREM2 has been shown to be involved in gastric, hepatocellular, and colorectal cancers, acting as a tumor promoter or suppressor [31–33]. In the current study, we found that TREM2 was associated with the PTC tumor microenvironment and further confirmed that the TREM2 expression was upregulated in PTC tissues. MTT and flow cytometry results confirmed that *TREM2* knockdown inhibited the proliferation and cell cycle of PTC cells, and transwell assays confirmed that *TREM2* knockdown inhibited cell migration and invasion. It is, thus, suggested that the antitumor effect of si-TREM2 may be related to the alteration of the tumor microenvironment.

However, our study had certain limitations. Bioinformatics analysis initially showed that TREM2 was differentially expressed in the PTC tumor microenvironment. However, whether TREM2 can influence the progression of PTC through regulation of TME effects needs further exploration. In addition, the biological function of TREM2 in PTC was only confirmed at the cellular level; these results should be validated in clinical and in vivo experiments. NF-*κ*B p65 is a protein factor that binds specifically to the immunoglobulin *κ* light chain gene enhancer *κ*B sequence. Combined with domestic and international reports, NF-*κ*B p65 can be involved in the development of thyroid cancer through various pathways, including suppression of the immune response, induction of cell proliferation, and prolongation of cell survival [34]. In the current study, KEGG enriched and analyzed 435 genes associated with the NF-*κ*B pathway, speculating that TREM2 may be involved in the immune microenvironment through the NF-*κ*B pathway. Previous studies have shown that TREM2 is involved in inflammatory injury by mediating the NF-*κ*B pathway [35]. In the current study, si-TREM2 inhibited the phosphorylation of NF-*κ*Bp65 and I*κ*B-*α* proteins and significantly declined the expression of p-NF-*κ*Bp65 and p-I*κ*B-*α*. It also downregulated the expression of proliferation-related protein cyclin D1 and antiapoptosis-related protein Bcl2 in the downstream pathway. It is suggested that TREM2 exerts a pro-proliferative effect on thyroid cancer cells by activating the NF-*κ*B signaling pathway to upregulate the expression of cyclin D1 and Bcl2, indicating that TREM2 may be located upstream of the NF-*κ*B signaling pathway. After blocking the expression of NF-*κ*B protein using different concentrations of PDTC, the expression of p-NF-*κ*Bp65 and p-I*κ*B-*α* protein was significantly downregulated with the gradual increase of PDTC dose. After blocking the NF-*κ*B pathway, the expression of TREM2 protein was also decreased. It is suggested that TREM2 may form a loop with NF-*κ*B signaling pathway.

In conclusion, in the current study, a series of analyses of DEGs arising from the comparison of the immune and stromal components of PTC revealed that possibly TREM2 was a potential indicator of altered TME status in PTC, and that TREM2 promoted PTC cell proliferation and cell cycle, migration, and invasion by NF-*κ*B pathway. This work provided new perspectives and directions for the diagnosis and treatment of PTC.

## Data Availability

The data used to support the findings of this study are available from the corresponding author upon request.
